# Algae as Protein Factories: Expression of a Human Antibody and the Respective Antigen in the Diatom *Phaeodactylum tricornutum*

**DOI:** 10.1371/journal.pone.0028424

**Published:** 2011-12-02

**Authors:** Franziska Hempel, Julia Lau, Andreas Klingl, Uwe G. Maier

**Affiliations:** 1 LOEWE Research Center for Synthetic Microbiology (SYNMIKRO), Marburg, Germany; 2 Laboratory for Cell Biology, Philipps University of Marburg, Marburg, Germany; Universidade de Sao Paulo, Brazil

## Abstract

Microalgae are thought to offer great potential as expression system for various industrial, therapeutic and diagnostic recombinant proteins as they combine high growth rates with all benefits of eukaryotic expression systems. Moreover, microalgae exhibit a phototrophic lifestyle like land plants, hence protein expression is fuelled by photosynthesis, which is CO_2_-neutral and involves only low production costs. So far, however, research on algal bioreactors for recombinant protein expression is very rare calling for further investigations in this highly promising field. In this study, we present data on the expression of a monoclonal human IgG antibody against the Hepatitis B surface protein and the respective antigen in the diatom *Phaeodactylum tricornutum*. Antibodies are fully-assembled and functional and accumulate to 8.7% of total soluble protein, which complies with 21 mg antibody per gram algal dry weight. The Hepatitis B surface protein is functional as well and is recognized by algae-produced and commercial antibodies.

## Introduction

Microalgae have been used in health food and cosmetic industry for a long time as they represent a natural source of lipids, vitamins, pigments and antioxidants [1]. The high oil content of certain species makes them additionally interesting for the biofuel industry as alternative to fossil fuels [2], [3], [4]. In recent years, microalgae came into biotechnical focus for yet another reason as they might show great potential as bioreactors for large-scale production of recombinant proteins. Microalgae combine high growth rates like prokaryotic cells with all advantages of eukaryotic expression systems, i.e. post-transcriptional and post-translational modifications and the assembly of multimeric protein complexes. Moreover, algae have a phototropic lifestyle hence their cultivation is CO_2_-neutral and involves comparatively low costs only – a great advantage to so far used expression systems like bacteria, yeast and mammalian or insect cells [1], [5], [6]. Diatoms like *Phaeodactylum tricornutum* are a group of algae with great ecological relevance as they are responsible for up to 20% of global CO_2_-fixation and are one of the most important sources of biomass in the oceans contributing to about 40% of marine primary production [7], [8]. In addition, diatoms represent an important source of lipids and silicate making them interesting for various biotechnological applications e.g. in biofuel industry, food industry and nanofabrication [9]. Moreover, a recent publication showed that a genetically manipulated diatom can produce the bioplastic PHB very efficiently [10]. Despite this progress in diatom biotechnology, however, diatoms have not been employed for expression of recombinant proteins with biotechnological relevance, hitherto.

Monoclonal antibodies are important tools in medical therapy, diagnostics and research and are mainly produced in mammalian cell lines, since the establishment of hybridoma technology in 1975 [11]. As cultivation of mammalian cells is very cost-intense, though, alternative expression systems are aspired and were tested in the past. Such include bacterial systems for the expression of antibody fragments as well as yeast, insect cells and transgenic plants [12], [13], [14], [15]. So far, however, none of these systems was able to substitute established mammalian expression systems with most critical limitations being low antibody expression levels, none or species-specific glycosylation, which is a critical factor for human therapy, or high production costs. Recently, a full-length IgG antibody was synthesized in the chloroplast of the green alga *Chlamydomonas reinhardtii* demonstrating that antibody expression in an algal system is feasible [16]. This finding certainly should entail further research, since algal systems show great potential as photosynthesis fuelled bioreactors for large-scale expression of therapeutic proteins like antibodies [17].

Vaccines are essential to prevent all kinds of severe infections, however high production costs limit vaccination especially in developing countries. The heterologous expression of antigen based vaccines is in most of the cases less complicated than antibody production, as eukaryotic expression systems are - depending on the respective protein - not necessarily required. However, the synthesis of antigens with complex post-translational modifications still needs cost-intensive mammalian expression systems bearing additionally a risk of human pathogenic contaminations. In this respect, microalgae offer great prospects as they are no host for human pathogens and combine fast growth rates with all advantages of eukaryotic expression systems. Once appropriate bioreactors are established, cultivation involves only low costs as light and water is almost all that is needed.

Hepatitis B is one of the most widespread viral infections with worldwide over 350 million people being chronic carriers [18]. Chronic Hepatitis B causes hepatic cirrhosis and hepatocellular carcinoma making an inexpensive therapy and vaccines essential [19]. A vaccine consisting of the Hepatitis B surface antigen (HBsAg) is available since 1982 and was formally isolated from high-titer patients. Today, HBsAg vaccine is produced in yeast [20], [21], but production and processing costs are still very high.

In this study, we present data on the synthesis of a fully-assembled and functional antibody against the Hepatitis B Virus surface protein in the diatom *P. tricornutum*. Furthermore, we demonstrate for the first time that the expression of the respective antigen is feasible in a microalgal expression system. Altogether, our studies highlight the enormous potential of a very efficient low cost and CO_2_-neutral expression system, which offers fast growth rates without the risk of human pathogenic contaminations.

## Results

### Expression of a fully-assembled human IgG antibody against HBsAg in *P. tricornutum*

**Figure 1:**
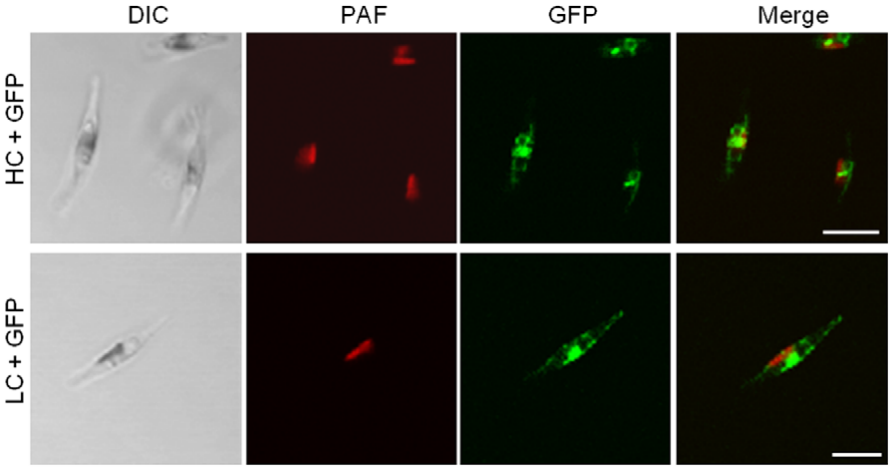
In vivo localization studies on antibody heavy and light chain expression in *P. tricornutum*. Localization studies demonstrate that heavy chain (HC) and light chain (LC) of the human IgG antibody CL4mAb fused to GFP accumulate in the endoplasmic reticulum of *P. tricornutum*. Hence, the endogenous signal peptide is sufficient to direct both antibody chains into the ER in the algal system. Scale bar represents 10 µm. PAF – plastid autofluorescence.

**Figure 2:**
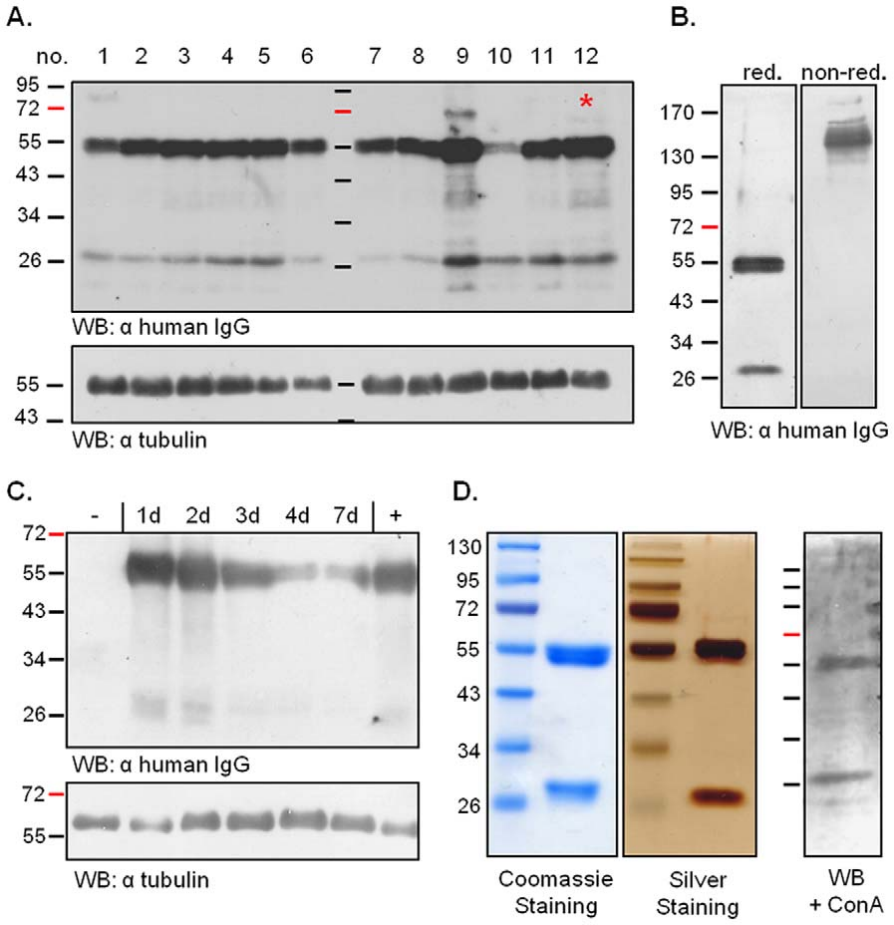
Analyses on antibody expression and assembly in *P. tricornutum*. **A.** Twelve independent *P. tricornutum* transfectants were screened for expression of heavy and light chain of the antibody. Western Blot analyses demonstrate that expression levels are very similar. For further analyses clone number 12 was selected as expression seems to be slightly enhanced. **B.** Western Blot analyses demonstrate that both antibody chains are expressed in *P. tricornutum* cells and assemble into complete antibodies. Under reduced conditions both antibody chains are detected separately, whereas in non-reduced SDS-PAGE a high molecular weight signal is detected, which corresponds to the fully-assembled antibody. **C.** Monoclonal antibodies are expressed under a NO_3_^−^-inducible promoter and different induction periods were tested to get highest expression levels. Best induction period turned out to be 1–2 days (lane 2+3), which was interestingly similar to expression levels with constant induction from the very beginning (lane 7). **D.** Antibodies expressed in *P. tricornutum* were purified using protein A-Sepharose beads. 5% of the eluate was loaded on a SDS-PAGE demonstrating that purification is very efficient with no contaminations detected by Coomassie or Silver Staining. Western Blot analyses with subsequent incubation with the lectin ConA revealed that both antibody chains get glycosylated in the ER of *P. tricornutum*. ConA – Concanavalin A, d – days.

The human monoclonal IgG1 antibody CL4mAb from cell-line TAPC301-CL4 recognizes the Hepatitis B surface antigen (HBsAg) and was selected for heterologous expression in *P. tricornutum* as it was already successfully expressed in other systems like tobacco and bacteria [22], [23]. Gene sequences for light and heavy chain (LC/HC) were adapted to *P. tricornutum* specific codon-usage and expressed as GFP fusion proteins in *P. tricornutum*. *In vivo* localization studies demonstrated that both antibody chains are expressed in the algal system and accumulate within the endoplasmic reticulum (Fig. 1). For expression of complete antibodies a co-transfection with sequences for both antibody chains was performed. An ER-retention signal (DDEL) was added at the C-terminus to avoid formation of complex glycosylation patterns within the Golgi apparatus, which might be a drawback in therapeutic applications [24]. In a small scale screening 12 independent transfectants were analysed for HC and LC expression by Western Blot analyses with an antibody against human IgG. Different transfectants showed slightly varying expression levels of HC and LC (Fig. 2A). For further analyses, the transfectant cell line with the highest expression level was selected and antibody assembly was inspected by non-reducing SDS-PAGE and Western Blot analyses. In contrast to reduced SDS-PAGE light and heavy chain were not detected separately but as high molecular weight signal of ∼160 kDa suggesting complete antibody assembly (Fig. 2B).

### Antibody quantification, purification and in vitro functionality analyses

For controlled expression of recombinant proteins in *P. tricornutum* an inducible system based on the nitrate reductase promoter/terminator was recently established with protein expression being easily controlled via NH_4_^+^/NO_3_^−^ supplements in the media [25]. Antibody expression was performed under inducible conditions to find best induction periods and circumvent potential growth defects due to over-expression. Best induction period turned out to be 1–2 days. After that time point antibody expression levels were significantly reduced (Fig. 2C). Interestingly, constant antibody expression by providing nitrate from the very beginning resulted in a high expression level similar to rates after 1–2 days (Fig. 2C). This demonstrates in addition that antibody accumulation does not impair growth rate and cell viability – a quite important attribute if large scale production is intended.

Quantification analyses demonstrated that antibody concentration lies at about 8.7% of total soluble protein on average. In absolute data this complies with approximately 400 µg antibody in a 250 ml culture, which needs about one week to reach adequate cell density. Antibody production in *P. tricornutum* was tested over 13 month with no decrease in antibody expression levels, thus antibody production seems to be stable for long terms. In general, *P. tricornutum* transfection has proven to be stable and because antibody expression is controlled by an inducible system – allowing non-induced stock preservation - there should be no problem with long-term stability of the production strains.

Antibody purification was carried out with protein A-Sepharose beads in small scale. Purification was very efficient with no contamination products or degradation products detected by Coomassie or Silver Staining (Fig. 2D). Further analyses revealed that both antibody chains get glycosylated in *P. tricornutum* as demonstrated by Western Blot analyses and incubation with lectins or subsequent oxidation with sodium-periodate, respectively (Fig. 2D).

**Figure 3:**
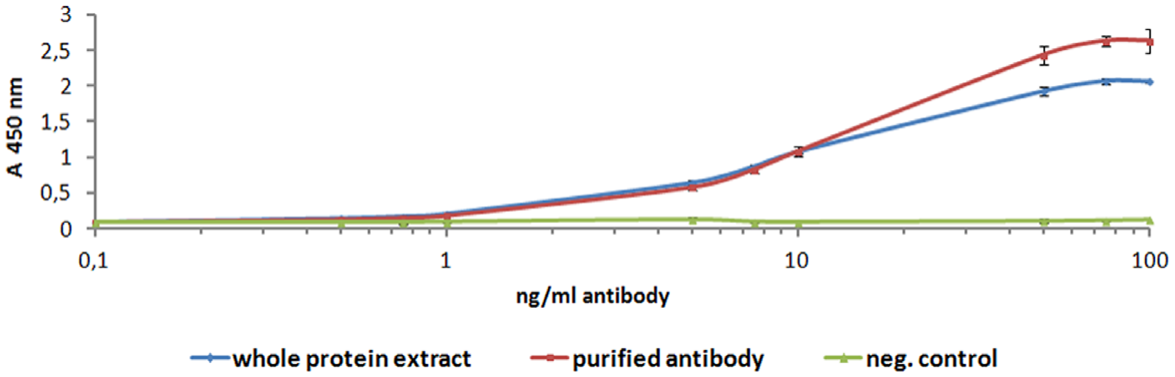
ELISA with antibodies against the Hepatitis B surface protein expressed in *P. tricornutum*. ELISA assays with purified antibody from *P. tricornutum* and whole cell extract of an antibody expressing clone demonstrate that algae-produced antibodies bind efficiently to commercial HBsAg reaching a binding maximum at 100 ng/ml. There was no antibody binding without HBsAg coated to the wells (negative control) or when using wild type protein extract (data not shown). Error bars indicate standard deviation (n = 3). The typical result of three experiments is shown.

Functionality of α-HBsAg antibodies produced in *P. tricornutum* was tested by ELISA. Wells were coated with Hepatitis B virus surface antigen and incubated with serially diluted purified antibody or whole protein extract of an antibody expressing clone, respectively. Thereby it was demonstrated that α-HBsAg antibodies produced in *P. tricornutum* are functional *in vitro* and reach a binding maximum at a concentration of 100 ng/ml (Fig. 3). Assays without antigen or with wild type protein extract served as negative control.

### Expression of the Hepatitis B virus surface antigen (HBsAg) in *P. tricornutum*

**Figure 4:**
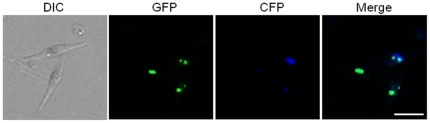
In vivo localization studies on Hepatitis B surface protein (HBsAg) expressed in P. tricornutum. HBsAg fused to GFP accumulates in punctual patterns, which partially co-localize with an ER-membrane protein (Der1-2) that was expressed as CFP fusion. The ER in *P. tricornutum* normally spans the whole cell, but is rather atypically aggregated in these clones. Punctual Fluorescence of HBsAg might be due to the formation of virus like particles (VLPs). Scale bar represents 10 µm. PAF – plastid autofluorescence.

The sequence of the Hepatitis B virus surface antigen (subtype *adr*) was adapted to *P. tricornutum* specific codon-usage and expressed as GFP fusion to check expression and localization in the algal system. *In vivo* localization studies demonstrate that the HBsAg GFP fusion proteins accumulate in several punctual structures within the cell. Co-localization studies with an ER resident membrane protein fused to CFP revealed that these structures are partially located within the ER as expected because of the N-terminal endogenous signal anchor sequence of HBsAg (Fig. 4). Additional punctual GFP accumulations might be due to the formation of virus like particles (VLP), which were observed in other expression systems like CHO cells [26], yeast [20], [21], [27] and also in plants [28], [29], [30].

For functionality analyses HBsAg was expressed without GFP but with an ER retention signal, which has shown to enhance HBsAg accumulation in plants [31]. Best induction time for HBsAg expression turned out to be 2 days with HBsAg expression levels of 0.7% of total soluble protein on average.

### Combined analyses on functionality of antibodies and antigens expressed in *P. tricornutum*

**Figure 5:**
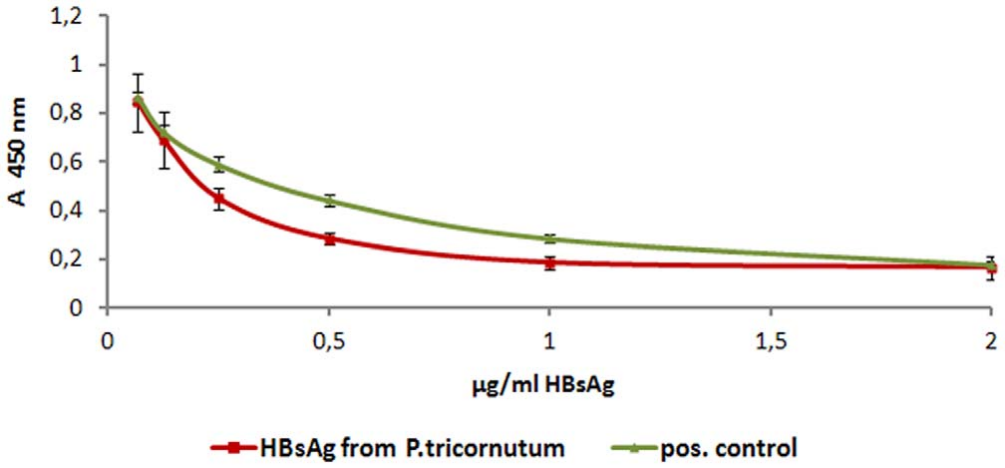
Inhibitory ELISA using algae-produced HBsAg and the respective algae-produced antibodies. Purified Anti-HBsAg antibody from *P. tricornutum* were mixed with serially diluted Hepatitis B surface protein (adr subtype) from *P. tricornutum* or *S. cerevisiae* (pos. control), respectively. HBsAg expressed in *P. tricornutum* inhibits antibody binding to solid-phase antigen slightly stronger than commercially available HBsAg from *S. cerevisiae*. Error bars indicate standard deviation (n = 3). The typical result of four experiments is shown.

Inhibitory ELISA was used to combine tests on *in vitro* functionality of antigen and antibody both being synthesized in the algal expression system. Antibodies against HBsAg were purified and incubated with serially diluted protein extract of HBsAg expressing cells to check on antibody potency for neutralizing HBV. Subsequently, the antigen/antibody mixture was added to HBsAg coated wells and antibody binding efficiency was measured. HBsAg expressed in *P. tricornutum* inhibited antibody binding proportional to antigen concentration even more effective than the positive control, which was commercially available HBsAg produced in *S. cerevisiae* (Fig. 5). In conclusion, the antibodies as well as the antigen expressed in *P. tricornutum* have proven to be fully functional *in vitro*.

## Discussion

Recombinant proteins are used in various industrial, therapeutic and diagnostic applications and consequently there is a great demand for expression systems involving low production costs. Well-established systems that are currently used for the majority of commercially available recombinant proteins are bacteria, yeast and mammalian bioreactors, however each system brings different limitations and the cost factor is still very high. Prokaryotic expression systems lack the ability of post-transcriptional and post-translation modifications, which are essential for the functionality of many eukaryotic proteins [24]. Yeast is not suitable as expression system for many mammalian proteins as multimeric protein assembly does not occur and glycosylation is in many cases incorrect [32]. Hence, mammalian systems still have to be used for the synthesis of many recombinant proteins, even though the expression level is low and mammalian bioreactors are difficult to handle and are thus very expensive [33].

In the late 1980s plants came into biotechnological focus as expression system for recombinant proteins, since they provide all advantages of higher eukaryotic expression systems but are fuelled by photosynthesis and hence do not need external carbon sources [34], [35], [36]. Thus, protein expression in plants involves low production costs and represents a CO_2_-neutral process. Another advantage to mammalian cell lines is the absence of human pathogenic contaminations making plants especially interesting for the expression of pharmaceutical proteins like antibodies, vaccines, hormones etc. [14], [37], [38], [39]. Indeed many projects show promising potential with some antibodies for example having reached clinical development stage (for review see Ref. 13). So far, however, plants are not established as biotechnological expression systems since protein expression levels and growth rates are still too low to make plant-produced protein expression profitable. Furthermore, plant-based expression systems depend on agricultural areas and spreading of transgenic plants is difficult to control shedding ethical concerns as well.

In this study, we tested the synthesis of an antibody and its respective antigen in an algal expression system - the diatom *P. tricornutum*. Microalgae provide all benefits of molecular farming in plants but offer many additional advantages since cultivation and handling is much easier and algae exhibit much higher growth rates, i.e. algae need only a few days to reach the biomass obtained by plants in several weeks [1], [5], [40]. Moreover, microalgae do not need agricultural areas but can be cultivated in special reactors like closed systems or open ponds [41], [42]. The results of this study demonstrate that the human antibody CL.4mAb against the Hepatitis B virus surface protein is expressed and assembled in the endoplasmatic reticulum of *P. tricornutum* and accumulates to approximately 8.7% of total soluble protein in two days of induction. When expressed in *Nicotiana tabacum* the same antibody reached much lower expression levels of only 0.2–0.6% in several weeks [22], indicating that expression in the algal system is much more efficient. Other antibodies against the Hepatits B virus produced in plants showed low expression levels (0.5%) as well [43]. Nevertheless, it is important to note that in other studies plant produced antibodies against different kinds of antigens reached expression levels of 0.1–6.5% of total soluble protein depending on various factors like plant species, plant organ, promoter choice, targeting signals etc. (for review see Ref. 13). One frequent problem with antibody expression in plants is rapid protein degradation resulting in low antibody levels and many fragmented products [44], [45], [46]. This does not seem to be a problem in *P. tricornutum* as both antibody chains were prominent with almost no degradation products being detected by Western Blot analyses. Antibody purification with protein A-Sepharose was carried out without problems, as neither antibody fragments, nor other contaminations were detected by Coomassie or Silver Staining. Finally, ELISA assays with whole protein extract as well as purified antibodies from *P. tricornutum* demonstrated that the algal-produced antibody is functional and binds the respective target antigen (HBsAg) very efficiently *in vitro*. A binding maximum is reached at concentrations comparable to the same antibody being expressed in *N. tabacum* and in the parental mammalian cell line, respectively [22].

A full-length IgG antibody against the anthrax antigen PA83 was previously expressed in an algal system - the green alga *C. reinhardtii* [16]. In this study, antibodies were expressed in the chloroplast, showing that antibody assembly is feasible in this formerly prokaryotic compartment as well. However, with 100 µg purified protein per gram dry cells the antibody expression level was not as efficient as in the ER of *P. tricornutum* were we found expression levels of 21 mg antibody per gram algal dry weight. In contrast to antibodies produced in the ER of *P. tricornutum*, the chloroplast expressed antibodies remained aglycosylated as this compartment lacks the respective equipment. Depending on the application this can also be an advantage, though [16].

In addition to antibody synthesis we tested the expression of the respective antigen, the Hepatitis B virus surface protein (HBsAg), in *P. tricornutum*. The HBsAg is commonly used as vaccine against Hepatitis B, however recombinant expression is complex and needs cost-intense eukaryotic expression systems since disulfide-bond formation as well as correct integration into the ER membrane have to occur [47], [48]. Again, plant-based bioreactors came into focus and first studies on expression of HBsAg in plants were performed in *N. tabacum* demonstrating that expression is possible in a plant system [29]. Many studies with agricultural crop like potato, banana and soybean confirmed that data and revealed the potential of oral immunization [30], [31], [49], [50]. Nevertheless, low expression levels, long growth periods and the need of agricultural areas prevented plant-based vaccination so far. In this study, we could show for the first time that HBsAg expression is possible in a microalgal system. HBsAg accumulated in *P. tricornutum* to 0.7% of total soluble protein, which is more efficient than in plant systems like *N. tabacum* showing expression levels of only 0.01–0.05% of total soluble protein [28], [29]. HBsAg expressed in *P. tricornutum* is recognized by commercially antibodies as well as by our algae-produced antibody as shown by ELISA and inhibitory ELISA assays.

Altogether, microalgae certainly offer great potential as low cost and CO_2_-neutral expression system for various recombinant proteins used in industrial, therapeutic and diagnostic applications. So far, however, only the green alga *C. reinhardtii* was employed for initial biotechnical approaches and other model organisms still need to be established. Our studies demonstrate that expression of a full-length IgG antibody against the Hepatitis B surface protein is very efficient in the diatom *P. tricornutum* with average expression levels of 9% of total soluble protein. Moreover, we could show that synthesis of complex antigens like the Hepatitis B surface protein is feasible in *P. tricornutum*. Since diatoms are in focus of diverse biotechnical applications like in fuel industry, food industry and nanotechnology, they could be interesting for combined approaches for example as expression system for pharmaceutics with lipids and silicate as side product.

## Materials and Methods

### Plasmid construction

DNA sequences for light and heavy chain of the monoclonal IgG1/kappa antibody CL4mAb (AF027158.2, AF027159.2) and the HBsAg subtype *adr* (AF504292) were adapted to *P. tricornutum* specific codon usage and synthesized by MrGene (Regensburg). Sequences of the synthetic constructs have been deposited in GenBank with accession numbers (JF970211, JF970210, JF970209). For *in vivo* localization studies sequences were cloned in front of eGFP into the vector pPha-NR, which is a derivate of pPhaT1 with endogenous nitrate reductase promoter/terminator flanking the multiple cloning site (JN180663). Co-localization studies of the HBsAg GFP fusion protein and the ER were performed with the ER membrane protein hDer1-2 (XP_002181749) fused to CFP. For expression of the fully-assembled antibody, DNA sequences for LC and HC were cloned into the vector pPha-DUAL[2×NR], which contains two multiple cloning sites both under the control of the endogenous nitrate reductase promoter (JN180664). For expression of active HBsAg used for ELISA, the sequence was cloned into pPha-NR without GFP fusion. To the C-terminus of antibody and antigen sequences an ER retention signal (DDEL) was added.

*P. tricornutum* (Bohlin, University of Texas Culture Collection, strain 646) was stably transfected by biolistic transfection as described previously [51] using M10 tungsten particles and 1350 psi rupture discs together with the particle delivery system Bio-Rad Biolistic PDS-1000/He. Cells were grown at 22°C under continuous illumination (80 µmol photons per m^2^ per s) on plates containing solid f/2-medium with 1.3% agar and 1.5 mM NH_4_^+^ as sole nitrogen source. Liquid cultures were grown with agitation (150 rpm) in 150–1000 ml erlenmeyer flasks to a density of about 7×10^6^ cells/ml.

### Expression and quantification of anti-HBsAg antibody and HBsAg

*P. tricornutum* cultures were grown under non-induced conditions in media containing 1.5 mM NH_4_^+^ and were then transferred to media containing 0.9 mM NO_3_^−^ for 1–2 days to induce expression of recombinant protein. For quantification of recombinant protein cells were harvested by centrifugation (5 min, 1.000 g) and resuspended in 5 ml buffer containing protease inhibitor cocktail. For analyses of antibody expression a buffer containing 0.1 M sodium phosphate pH 7.4 and 0.15 M NaCl was used. For HBsAg quantification the buffer contained PBS pH 7.2, 5 mg/ml sodium ascorbate, 10 mM EDTA and 0.1% TritonX-100. Cells were disrupted using a French press (20.000 psi cell pressure, five repeats) and cell debris was removed by centrifugation (30 min, 20.000 g). For solubilization of HBsAg the protein extract was incubated for 2 h with agitation before removal of insoluble material. Total soluble protein was determined by standard Bradford assays. For antibody quantification the Easy-Titer human IgG (H+L) assay kit (ThermoScientific) was used with human IgG as standard (550 ng/ml–17.2 ng/ml). Total soluble protein extract or purified antibody was serially diluted and antibody concentration was measured according to manufacturer's instructions. Wild type algal protein extract served as negative control. To determine antibody portions per algal dry weight a *P. tricornutum* culture was split with half of the cells being lyophilized and weighted and half being used for quantification. For quantification of HBsAg the Hepatitis B surface antigen ELISA kit (Anogen) was used with HBsAg *adr* protein (Abcam) as standard (550 ng/ml–34.4 ng/ml). Total soluble protein extract of *P. tricornutum* transformants was serially diluted and HBsAg concentration was determined according to manufacturer's instructions with wild type protein extract as negative control.

### Purification of anti-HBsAg antibody

Protein extract of 250 ml *P. tricornutum* culture was prepared as described above and incubated for 3 h at 4°C with 400 µl protein A-Sepharose beads. After washing with purification buffer, anti-HBsAg antibody was eluted with 0.2 M Glycin/HCl pH 2.5 and neutralized with 1 M Tris/HCl pH 9. Purification and antibody assembly was inspected by SDS-PAGE and Western Blot analyses under reducing (5% β-mercaptoethanol) and non-reducing (no β-mercaptoethanol) conditions. In Western Blot analyses biotin-labelled anti- human IgG antibody was used and detected with Streptavidin HRP. Glycosylation of antibody chains was checked by incubation with ConA-HRP.

### ELISA

ELISA plates were coated with 200 ng/well HBsAg subtype *adr* (Abcam) in 50 mM NaHCO_3_ pH 8.3, washed with PBS+0.1% Tween-20 and blocked with 1% BSA in PBS. After washing, 50 µl serially diluted algal protein extract or purified antibody was added for 2 h. Wells were washed again and incubated with HRP coupled α-human IgG antibody for 1 h. After final washing bound antibodies were detected using POD substrate. For inhibitory ELISA plates were coated with HBsAg, washed and blocked as described above. Protein extract of *P. tricornutum* cells expressing HBsAg was serially diluted to HBsAg concentrations of 2 µg/ml–62.5 ng/ml. 50 µl were mixed with equal volume of purified anti-HBsAg antibody (50 ng/ml) from *P. tricornutum* and incubated for 1 h. Subsequently, 50 µl of the antigen-antibody mixture was added to the wells and incubated for 2 h. Detection of bound antibody was handled as described above. Commercially available HBsAg was serially diluted and served as positive control in a wild type protein extract background.

### Fluorescence microscopy

*In vivo* localization of GFP fusion proteins was analysed with confocal laser scanning microscopes Leica TCS SP2 and SP5 using a HCX PLAPO 63×/1.32–0.6 oil Ph3 CS objective. GFP and chlorophyll fluorescence was excited at 488 nm and detected at a bandwidth of 500–520 nm and 625–720 nm, respectively. CFP fluorescence was excited at 405 nm and detected with a bandwidth of 430–480 nm.
